# Indications for Corneal Transplantation at a Tertiary Referral Center in Tehran

**Published:** 2010-04

**Authors:** Mohammad Zare, Mohammad-Ali Javadi, Bahram Einollahi, Alireza Baradaran-Rafii, Siamak Zarei Ghanavati, Mohammad-Reza Jamshidi Farsani, Parviz Mohammadi, Sepehr Feizi

**Affiliations:** Ophthalmic Research Center, Labbafinejad Medical Center, Shahid Beheshti University of Medical Sciences, Tehran, Iran

**Keywords:** Corneal Transplantation, Penetrating Keratoplasty, Lamellar Keratoplasty

## Abstract

**Purpose:**

To report the indications and techniques of corneal transplantation at a tertiary referral center in Tehran over a 3-year period.

**Methods:**

Records of patients who had undergone any kind of corneal transplantation at Labbafinejad Medical Center, Tehran, Iran from March 2004 to March 2007 were reviewed to determine the indications and types of corneal transplantation.

**Results:**

During this period, 776 eyes of 756 patients (including 504 male subjects) with mean age of 41.3±21.3 years underwent corneal transplantation. The most common indication was keratoconus (n=317, 40.8%) followed by bullous keratopathy (n=90, 11.6%), non-herpetic corneal scars (n=62, 8.0%), infectious corneal ulcers (n=61, 7.9%), previously failed grafts (n=61, 7.9%), endothelial and stromal corneal dystrophies (n=28, 3.6%), and trachoma keratopathy (n=26, 3.3%). Other indications including Terrien’s marginal degeneration, post-LASIK keratectasia, trauma, chemical burns, and peripheral ulcerative keratitis constituted the rest of cases. Techniques of corneal transplantation included penetrating keratoplasty (n=607, 78.2%), deep anterior lamellar keratoplasty (n=108, 13.9%), conventional lamellar keratoplasty (n=44, 5.7%), automated lamellar therapeutic keratoplasty (n=8, 1.0%), and Descemet stripping endothelial keratoplasty (n=6, 0.8%) in descending order. The remaining cases were endothelial keratoplasty and sclerokeratoplasty.

**Conclusion:**

In this study, keratoconus was the most common indication for penetrating keratoplasty which was the most prevalent technique of corneal transplantation. However, deep anterior lamellar keratoplasty is emerging as a growing alternative for corneal pathologies not involving the endothelium.

## INTRODUCTION

Corneal blindness is a major health problem.^1^ The epidemiology of corneal pathologies in each region facilitates the allocation of resources. Certain indications for penetrating keratoplasty have been changing in Iran.^2^ Furthermore, the technique of corneal transplantation has evolved over time and new procedures including deep anterior lamellar keratoplasty (DALK) and Descemet stripping endothelial keratoplasty (DSEK) have been recently introduced. Therefore, it is necessary to revise the data on the prevalence of corneal pathologies and techniques of corneal transplantation.

Herein, we report the indications and techniques of corneal transplantation performed at Labbafinejad Medical Center, a tertiary referral center in Tehran, Iran over a 3-year period to determine any change in the frequency and indications of corneal transplantation and trends in surgical techniques during this period.

## METHODS

In this retrospective study, hospital records of patients who had undergone corneal transplantation at Labbafinejad Medical Center from March 2004 to March 2007 were reviewed for demographic data, indications for keratoplasty and surgical technique. Indications for keratoplasty were the clinical diagnosis made by the surgeon at the time of the operation. When multiple transplantations were performed, the diagnosis was considered to be “regraft” regardless of the initial indication for transplantation and the type of keratoplasty. Aphakic or pseudophakic patients who underwent keratoplasty for corneal decompensation were considered to have aphakic bullous keratopathy (ABK) or pseudophakic bullous keratopathy (PBK), regardless of the underlying mechanism of corneal decompensation.

## RESULTS

**Figure 1:**
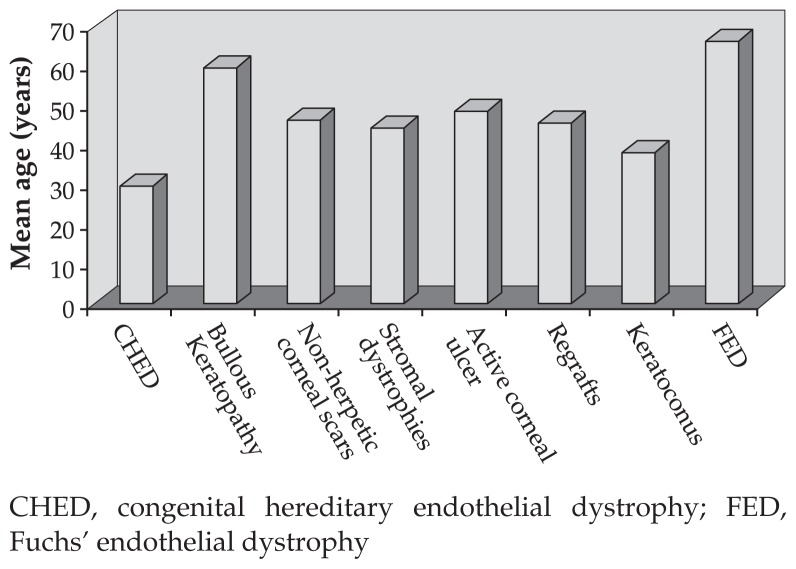
Mean age of patients in each subgroup of corneal pathologies. CHED, congenital hereditary endothelial dystrophy; FED, Fuchs’ endothelial dystrophy

Overall, 776 eyes of 756 patients including 504 (64.9%) male and 252 (35.1%) female subjects underwent corneal transplantation during the period of the study. Mean age was 41.3±21.3 years (range, 10 days to 89 years) with a median of 39 years. As shown in Figure 1, patients with congenital hereditary endothelial dystrophy (CHED) were the youngest (mean age, 31.7 years) and subjects with Fuchs’ endothelial dystrophy (FED) were the oldest (mean age, 68.4 years) patients.

The most common indication for keratoplasty was keratoconus (n=317, 40.8%) followed by bullous keratopathy (n=90, 11.6%), non-herpetic corneal scars (n=62, 8.0%), infectious corneal ulcers (n=61, 7.9%), previously failed grafts (n=61, 7.9%), endothelial and stromal corneal dystrophies (n=28, 3.6%), and trachoma keratopathy (n=26, 3.3%). The remaining indications (n=131, 16.9%) included Terrien’s marginal degeneration, post-LASIK keratectasia, trauma, chemical burns, and peripheral ulcerative keratitis.

**Figure 2:**
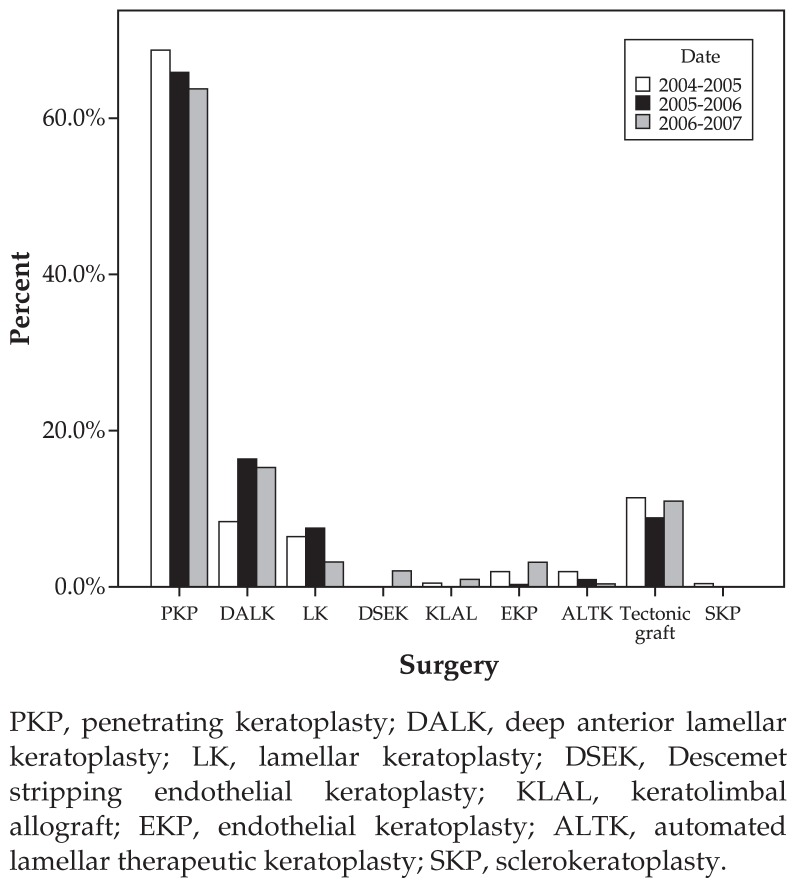
Techniques of keratoplasty during the study period. PKP, penetrating keratoplasty; DALK, deep anterior lamellar keratoplasty; LK, lamellar keratoplasty; DSEK, Descemet stripping endothelial keratoplasty; KLAL, keratolimbal allograft; EKP, endothelial keratoplasty; ALTK, automated lamellar therapeutic keratoplasty; SKP, sclerokeratoplasty.

Techniques of corneal transplantations included penetrating keratoplasty (PKP; n=607, 78.2%), DALK (n=108, 13.9%), lamellar keratoplasty (LK; n=44, 5.7%), automated lamellar therapeutic keratoplasty (ALTK; n=8, 1.0%), and Descemet stripping endothelial keratoplasty (DSEK; n=6, 0.8%) in descending order. The remaining methods included endothelial keratoplasty and sclerokeratoplasty (Fig. 2).

Of 61 eyes receiving PKP for management of infectious corneal ulcers, 29 (47.5%) had bacterial keratitis and 28 (45.9%) had fungal keratitis. Only one (1.6%) eye had culture-positive acanthamoeba keratitis and the rest remained undiagnosed.

## DISCUSSION

A report by Kanavi et al^2^ on the indications for PKP in Iran between 1997 and 2003 showed that the most common indication was keratoconus followed by corneal opacities and scars, PBK, corneal dystrophies, ABK, and regrafts in descending order. In the current study, the leading indication for corneal transplantation was still keratoconus but bullous keratopathy constituted the second most common indication; non-herpetic corneal scars and infectious corneal ulcers ranked third and fourth, respectively. Stromal and endothelial corneal dystrophies were the least common indications. This alteration may reflect a recent change in indications for corneal transplantation in the country as our center is a tertiary referral center and deals with patients referred from all over the country.

Keratoconus constitutes the most common indication for corneal transplantation in some countries.^2^–^7^ Genetic and environmental factors, alternative therapeutic approaches such as contact lens fitting, access to donor cornea tissue, and cost of surgery may influence this issue.^8^,^9^

There is no report on the prevalence of keratoconus in Iran, but it appears to be relatively high. One explanation may be the association between keratoconus and vernal keratoconjunctivitis which is very common in Iran.^2^ The fact that the leading indication for corneal transplantation is keratoconus can be explained by easy access to donor corneas procured from the Eye Bank of the Islamic Republic of Iran leading to early operation especially with the recent resurgence of interest in DALK.

Regraft is the most common indication for corneal transplantation in Great Britain,^10^ however bullous keratopathy was the most common indication in the USA.^11^ In some developing countries such as India and China, corneal infections were the leading cause of keratoplasty between 1997 and 2002.^4^,^9^,^12^

Compared to previous reports from this center,^13^,^14^ the incidence of bullous keratopathy rose from third place to second; bullous keratopathy ranked third in the country between 1984 and 1994.^13^ The majority of cases of bullous keratopathy included pseudophakic patients (86.8%). Such an increase can be attributed to the shift in the technique of cataract surgery from extracapsular extraction to phacoemulsification with more damage to endothelial cells.^15^,^16^

Non-herpetic corneal scars ranked third in our study, while in some developing countries it is the leading indication for corneal transplantation (ranging from 27.9% to 38.0%).^17^–^21^ The underlying causes of corneal scar such as trauma, corneal ulcers, and trachoma keratopathy were apparent in a small proportion of patients. Trachoma keratopathy which used to be one of the leading indications for corneal transplantation has dramatically decreased, thanks to improved sanitation.^22^

A striking finding was the low incidence of regrafts in our study which is in line with in nationwide reports^2^ (7.9% and 5.2%, respectively) as compared to the USA^11^ and Canada.^17^ Dobbins et al^11^ have found an increasing trend in the incidence of regrafts which can be related to an increasing rate of corneal transplantation. The lower incidence of regrafts in our study can be explained by good quality grafts harvested from young donors.

Many studies conducted in western countries reported FED as an important indication with a rate of 9.3% to 23.2%.^7^,^11^ However it was a rare condition in the present study (0.5%) and other studies performed in Iran (1.7%).^13^,^14^ Macular corneal dystrophy was the most common dystrophy requiring corneal transplantation in the current study. This finding supports the report by Kanavi et al.^2^ Active infectious keratitis was an uncommon condition in this report, leading to corneal transplantation in 10.1%, of which 37.2% were caused by bacterial agents, 37.2% by fungal agents, 24.4% by herpes simplex virus, and 1.3% by acanthamoeba.

In countries with large rural communities such as China and India, active infectious keratitis caused by fungal agents is the leading indication for corneal transplantation. This indicates how difficult it is to prevent and treat infectious corneal ulcers in developing countries.^4^,^9^,^15^

In the current series, penetrating keratoplasty was the most common technique of corneal transplantation (78.2%) followed by DALK, which increased from 17 cases in 2004 to 48 cases in 2005 and 43 cases in 2006, summing up to 13.9% of all procedures during the period of the study. DALK, which was only performed in eyes with keratoconus in the current study, eliminates the complications encountered during open-sky surgery (PKP) and endothelial graft rejection reactions.^23^

Tectonic grafts (89 eyes, 10.3%) were separately reported in this series. Other techniques presented in descending order were conventional LK (5.7%), ALTK (1.0%), KLAL (keratolimbal allograft, 0.6%), and DSEK (0.8%).

At our center, DSEK was performed for the first time in 2006. Since this technique is associated with fast visual rehabilitation and less postoperative morbidity, it has the potential to become the preferred technique of transplantation for bullous keratopathy and FED.

Median age in this study was 39 years which can be explained by the large number of young patients with keratoconus who constituted the majority of participants. Subjects with CHED were the youngest and patients with PBK were the oldest.

In conclusion, keratoconus remains the leading indication for keratoplasty, resembling previous reports from Iran. As compared to a previous report, the incidence of bullous keratopathy has increased probably due to the shift in cataract surgery technique from extracapsular cataract extraction to phacoemulsification. DALK has emerged as an alternative for PK in keratoconic eyes and DSEK has also been increasingly employed for eyes with an abnormal endothelium.

## References

[1] Maurin JF, Cornand G (1990). Corneal blindness in tropical environment. Rev Int Trach Pathol Ocul Trop Subtrop Sante Publique.

[2] Kanavi MR, Javadi MA, Sanagoo M (2007). Indications for penetrating keratoplasty in Iran. Cornea.

[3] Flowers CW, Chanq KY, McLeod SD, Irvine JA, McDonnell PJ, Rao N (1995). Changing indications for penetrating keratoplasty, 1989–1993. Cornea.

[4] Sony P, Sharma N, Sen S, Vajpayee RB (2005). Indications of penetrating keratoplasty in Northern India. Cornea.

[5] Cosar CB, Sridhar MS, Cohen EJ, Held EL, Alvim Pde T, Rapuano CJ (2002). Indications for penetrating keratoplasty and associated procedures, 1996–2000. Cornea.

[6] Liu E, Slomovic AR (1997). Indications for penetrating keratoplasty in Canada, 1986–1995. Cornea.

[7] Edwards M, Clover GM, Brookes N, Pendergrast D, Chaulk J, McGhee CN (2002). Indications for corneal transplantation in New Zealand: 1991–1999. Cornea.

[8] Chen WL, Hu FR, Wang IJ (2001). Changing indications for penetrating keratoplasty in Taiwan from 1987 to 1999. Cornea.

[9] Dandona L, Ragu K, Janarthanan M, Naduvilath TJ, Shenoy R, Rao GN (1997). Indications for penetrating keratoplasty in India. Indian J Ophthalmol.

[10] Al-Yousuf N, Mavrikakis I, Mavrikakis E, Daya SM (2004). Penetrating keratoplasty: indications over a 10 year period. Br J Ophthalmol.

[11] Dobbins KR, Price FW, Whitson WE (2000). Trends in the indications for penetrating keratoplasty in the midwestern United States. Cornea.

[12] Zhang C, Xu J (2005). Indications for penetrating keratoplasty in East China, 1994–2003. Graefes Arch Clin Exp Ophthalmol.

[13] Zare M, Norouzizadeh M, Javadi MA, Karimian F, Einollahi B, Sajjadi H (1998). Evaluation of corneal transplantation and its outcomes in Labbafinejad Medical Center between 1986 and 1993. Bina J Ophthalmol.

[14] Soleimani M, Javadi MA, Zare M, Sharifi A (2005). Indications for corneal transplantation in Labbafinejad Medical Center, 2001–2002. Bina J Ophthalmol.

[15] Xie L, Song Z, Zhao J, Shi W, Wang F (2007). Indications for penetrating keratoplasty in north China. Cornea.

[16] Claesson M, Armitage WJ, Stenevi U (2009). Corneal oedema after cataract surgery: predisposing factors and corneal graft outcome. Acta Ophthalmol.

[17] Damji KF, Rootman J, White VA, Dubord PJ, Richards JS (1990). Changing indications for penetrating keratoplasty in Vancouver, 1978–1987. Can J Ophthalmol.

[18] Kervick GN, Shepherd WF (1990). Changing indications for penetrating keratoplasty. Ophthalmic Surg.

[19] De Cock R (1994). Penetrating keratoplasty in the West Bank and Gaza. Eye (Lond).

[20] Lang GK, Wilk CM, Naumann GO (1988). Changes in the indications status for keratoplasty (Erlangen, 1964–1986). Fortschr Ophthalmol.

[21] Maeno A, Naor J, Lee HM, Hunter WS, Rootman DS (2000). Three decades of corneal transplantation: indications and patient characteristics. Cornea.

[22] Zare M, Aghadoost D, Delavari A, Mostafaii G, Hajijafari M, Parsikia A (2006). Rapid assessment of trachoma (RAT) in south provinces of Iran. Bina J Ophthalmol.

[23] Watson SL, Ramsay A, Dart JK, Bunce C, Craig E (2004). Comparison of deep lamellar keratoplasty and penetrating keratoplasty in patients with keratoconus. Ophthalmology.

